# Influence on Therapeutic Decision Making of SPECT-CT for Different Regions of the Foot and Ankle

**DOI:** 10.1155/2014/927576

**Published:** 2014-05-18

**Authors:** Leif Claassen, Theodor Uden, Max Ettinger, Kiriakos Daniilidis, Christina Stukenborg-Colsman, Christian Plaass

**Affiliations:** Hannover Medical School, Orthopedic Department, Anna-von-Borries-Straße 1-7, 30625 Hannover, Germany

## Abstract

*Background*. Single-photon emission computed tomography and computed tomography (SPECT-CT) has a high impact on diagnosis and treatment decision of different joints. The aim of this study was to evaluate whether there is a different gain of SPECT-CT for different foot regions. *Material and Methods*. We retrospectively identified 86 patients who received a SPECT-CT of the foot and ankle between April 2011 and December 2012. We divided all patients into the following subgroups: ankle (group 1), subtalar (group 2), Chopart (group 3), and Lisfranc (group 4). The local ethical committee approved the study. *Results*. The clinical treatment decision was changed based on SPECT-CT results in 64.5% of group 1, 65.2% of group 2, and 75% each of groups 3 and 4. Eighty patients (93%) had pain relief after treatment based on SPECT-CT. The overall SPECT-CT sensitivity was 0.94 and the specificity was 0.57. The positive and negative predictive values were 0.87 and 0.75, respectively. *Conclusion*. The impact of SPECT-CT on treatment decision is slightly higher in diseases of the Chopart and Lisfranc joints than in the upper ankle and subtalar joints. The additional information has a clinical relevance due to the high rate of pain relief by treatment based on SPECT-CT diagnosis.

## 1. Introduction

In foot and ankle surgery a diagnostic uncertainty may remain despite a thorough radiological and clinical examination. The main reason is the complex anatomy with proximity of many small joints [1, 2]. This applies notably for Chopart joint and even more for Lisfranc joint [3]. The expanded diagnosis with MRI and CT scans offers morphological information [1, 2].  MRI has developed to the leading imaging modality amongst others due to a well definition of soft tissue and increased quality of bone imaging [4]. Still an unclear diagnosis can persist due to missing functional data. The morphological changes often do not contribute to the patients' symptoms, or the pathologies did not lead to morphological changes yet. Bone scan is used to evaluate bony pathologies since the 1960's analyzing the functional activity of pathology [4]. While bone scan alone has a lack of spatial resolution, the combination of bone scan and CT offers morphological and functional data [2, 5–7]. Common indications in foot and ankle surgery are exact localization of arthritis, osteochondrosis dissecans, coalitio, stress fractures, and sesamoiditis [8–11]. The advantages of SPECT-CT led to an increasing use [2, 6, 8]. Scharf assumed that, within orthopedic practice, SPECT-CT has the biggest impact in foot and ankle surgery [4]. The present study was performed to analyze a possible different value of SPECT-CT in hindfoot and midfoot. Our hypothesis was that the effect of SPECT-CT on diagnosis, treatment decision, and treatment outcome based on SPECT-CT is higher in Chopart and Lisfranc joints than in ankle and subtalar joints.

## 2. Material and Methods

We performed a study in our level-one foot and ankle center on 86 patients with complex foot pathologies who received a SPECT-CT as sole or additional examination of the foot and ankle between April 2011 and December 2012.  Inclusion criteria were a SPECT-CT prior to intervention and no loss to followup. Patients who did not adhere to treatment recommendation were excluded.  The local ethical committee approved the study. The mean age was 54 ± 15.3 years. Thirty-five patients were male and 51 female.

**Figure 1:**
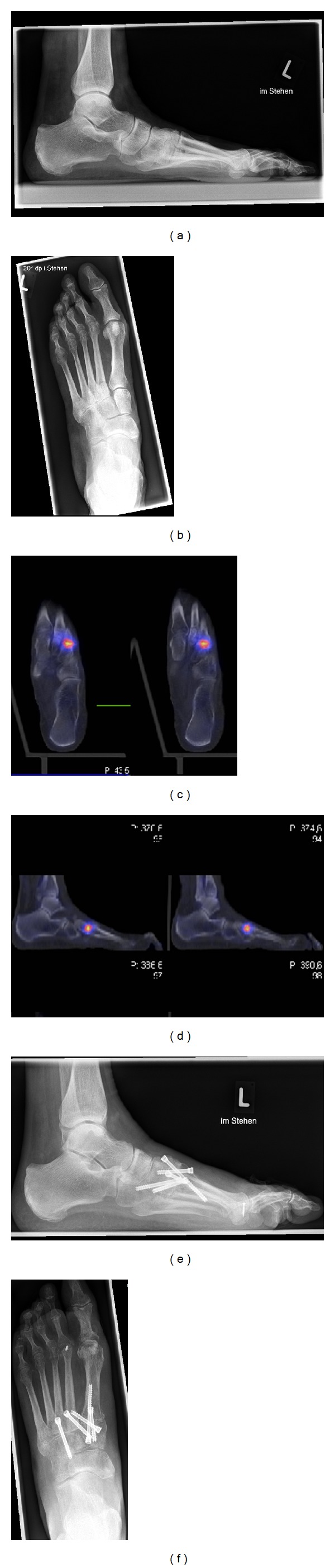
(a) and (b) preoperative plain radiographs; (c) and (d) SPECT-CT; (e) and (f) postoperative plain radiographs. A 63-year-old woman with pes plano-valgus deformity described pain in the whole foot for many years. A conservative treatment with insoles and physiotherapy failed. After clinical examination and plane radiographs subtalar arthritis was the assumed diagnosis. Because of diagnostic uncertainty a SPECT-CT was performed resulting in an active arthritis of TMT II and III and arthritis of TMT-I. After fusion of TMT I-III the patient described pain relief five months after the operation.

Prior to performing a SPECT-CT we conducted a thorough examination including clinical assessment and radiographs in at least two planes. Indication for SPECT-CT was inconclusive findings in conventional clinical and radiological assessment (Figure 1).

We performed a triple phase bone scan of the feet with 500 MBq of Technetium-99m hydroxymethylene disphosphonate (99mTc-HDP) that was injected intravenously and acquired with a Symbia T2 SPECT-CT scanner (Siemens Healthcare, Erlangen, Germany). Acquiring blood pool images over three minutes and lateral images over four minutes followed a perfusion phase of three minutes. After an intermission of two hours late phase images were performed. Subsequently CT component was conducted with 120 kV and 25 mAs. The images were reconstructed with one millimeter slice thickness. Two experienced radiologists analyzed the SPECT-CT.

The diagnostic decision was based on pathologies visible with the CT (e.g., joint space narrowing, osteophytic lipping, subchondral sclerosis and cysts, osteonecrosis, and fracture) combined with increased metabolism of the bone.

The performed operation or, in cases of conservative treatment, the main diagnosed pathology was decisive to divide the patients into four groups—patients with main pathology in the ankle (group 1), subtalar (group 2), Chopart (group 3), and Lisfranc (group 4). We evaluated the rate of changed diagnosis from initial consultation after completion of standard assessment compared to diagnosis and treatment based on SPECT-CT. We determined the sensitivity, specificity, and positive and negative predictive values for each group.

Additionally we evaluated patient's pain level via the visual analogue scale (VAS) prior to operation. All patients were subsequently followed up to analyze the postoperative pain level via VAS. Patients who had a pain relief of more than 50% were considered to have a successful outcome [6].

The data collection and statistical analysis were performed with GraphPad Prism 5 (GraphPad Software, Inc., La Jolla, CA). The values are expressed as mean with 95% confidence interval. We evaluated the sensitivity, specificity, and positive and negative predictive value. The comparisons between the values of the different groups were performed with the Fisher's exact test. A statistical analysis of symptomatic improvement, changed treatment decision, and rate of operative treatment was not possible.

## 3. Results

Thirty-one patients (36%) were classified for group 1, 23 patients (26%) for group 2, and 16 patients each (19%) for groups 3 and 4. The overall sensitivity for all patients was 0.94, the specificity was 0.57. The positive and negative predictive values were 0.87 and 0.75, respectively.

In group 1 the diagnosis was changed in 20/31 patients (64.5%). 23/31 patients were operated (74.2%) and 28/31 patients had a pain relief of at least 50% after treatment (90.3%) (Table 1).  For group 1 we found a sensitivity of 0.89 (0.71–0.98), a specificity of 0.67 (0.09–0.99), and positive and negative predictive values of 0.96 (0.80–1.0) and 0.4 (0.05–0.85). The three patients with persistent pain were treated operatively (Table 2).

In 15/23 cases of group 2 the treatment was changed due to SPECT-CT (65.2%). 18/23 patients received an operation (78.3%) and 22/23 patients described a pain relief after treatment (95.7%) (Table 1). The sensitivity for group 2 was 0.94 (0.73–1.0), the specificity was 0.80 (0.28–0.99). The positive predictive value was 0.94 (0.73–1.0) and the negative predictive value was 0.80 (0.28–0.99). The patient with persistent pain was treated conservatively (Table 2).

We found a changed diagnosis in 12/16 patients of group 3 (75%). 9/16 patients were treated operatively (56.2%) and 15/16 patients had a pain relief after treatment (93.8%) (Table 1). For group 3 the data resulted in a sensitivity of 1.0 (0.63–1.0), a specificity of 0.63 (0.24–0.91), and positive and negative predictive values of 0.73 (0.39–0.94) and 1.0 (0.48–1.0). The patient without pain relief was treated conservatively (Table 2).

The diagnosis and treatment were altered in 12/16 patients of group 4 based on SPECT-CT findings (75%). 11/16 patients were treated operatively (68.8%) and 15/16 patients had a pain relief after treatment (93.8%) (Table 1). For group 4 the data resulted in a sensitivity of 1.0 (0.72–1.0),  a specificity of 0.20 (0.01–0.72),  and positive and negative predictive values of 0.73 (0.45–0.92) and 1.0 (0.03–1.0). The patient without pain relief was treated operatively (Table 2).

The comparison of sensitivity, specificity, and positive and negative predictive value showed no significant differences. Conflating ankle and subtalar groups and Chopart and Lisfranc groups did not change this fact.

The rates of changed diagnosis and treatment as well as the rate of symptomatic improvement are slightly higher in groups 3 and 4 compared to groups 1 and 2.

## 4. Discussion

This study was performed to itemize the known impact of SPECT-CT on treatment decision in foot and ankle surgery to different foot regions. We could demonstrate that SPECT-CT leads to a high rate of altered treatment decision in foot and ankle surgery, especially in Lisfranc and Chopart joint lines.

The presented study has some limitations. We had no control group and the study was performed retrospectively. Additionally the number of cases is limited.

Primary diagnostic in foot and ankle surgery consists of clinical examination and plain radiographs. In case of doubt sonography, CT scan or MRI can be performed to verify the assumed or exclude differential diagnosis [6]. Nevertheless in some cases a diagnostic ambiguity can persist. One reason might be that MRI and sonography have advantages in soft tissue but low specificity for bony pathologies [4]. CT scan can illustrate bony pathologies with a high resolution but still there is missing information about the activity of the pathology. For complex and difficult cases SPECT-CT has been widely described as an adjuvant and effective tool to reach a diagnosis [2, 3, 6, 8–11].

In the majority of patients in foot and ankle surgery bony pathologies are more likely to be the cause of pain compared to soft tissue disorders. For osteochondral defects, for example, it has been shown by van Dijk et al. that the pain source is based on the subchondral bone and not on the cartilage [12]. Hence we think that findings in SPECT-CT have a well correlation with the pain generation as it illustrates the bone metabolism. In the present study for treatment decision the metabolic activity was concordantly judged as superior to morphologic changes due to the assumption that severe deformities could be asymptomatic while already little changes with high metabolic activities might be symptomatic [13].

SPECT is based on the detection of gamma rays that are emitted by a radionuclide injected into the patient's blood stream. Therefore, ^99m^Tc-labelled phosphates are often used. It is enhanced in regions of increased bone turnover due to an enhanced vascularity and osteoblastic presence [6, 11, 14]. The usefulness of SPECT alone is limited through a low spatial resolution and missing anatomical markers [11]. These problems are solved through its combination with a high resolution CT scan. SPECT-CT has an increasing relevance in diagnosis of several medical specialties [4, 7]. Moreover several authors described its importance in foot and ankle surgery [2, 4, 6, 9–11, 14]. In matter of soft tissue pathologies of the foot there are different recommendations whether to use SPECT-CT. Where Huellner and Strobel describe usefulness Singh et al. would deny the relevance of SPECT-CT in soft tissue pathologies [6, 10]. For bony pathologies Pagenstert et al. could verify high intraobserver and interobserver reliability for SPECT-CT. It was higher in comparison to bone scan and CT scan alone [2]. Nathan et al. described in a case series additional information through SPECT-CT in 25 of 31 patients (81%) and changed management in 62% [11]. Scharf appraises a main domain of SPECT-CT in foot and ankle surgery in the assessment of sport trauma. Here SPECT-CT might be beneficial through confining old from new traumata [4]. In a most recent study of Singh et al. the treatment was changed in 39 of 50 patients (78%) after performing a SPECT-CT. Additionally 46 of 50 patients (92%) had a symptomatic improvement after treatment based on SPECT-CT [6]. But although several authors associated the special importance of SPECT-CT in foot and ankle surgery with the proximity of anatomical structures evidence for different impact of SPECT-CT for different foot regions is missing.

In our study we could affirm the meaning of SPECT-CT in foot and ankle surgery. The treatment decision was changed in 68.6%; 93% of all patients had a symptomatic improvement. This corresponds to the published data of Singh et al. and Nathan et al. [6, 11]. Concerning the high rate of changed treatment decision in our study it is important to stress that patients receiving a SPECT-CT represent the most complex and difficult to diagnose cases. The 86 patients represent less than 10% of patients treated during that time in our tertiary care foot and ankle department. It has to be emphasized that the high rate of changed diagnosis and treatment decision is probably due to the selected complex cases, where SPECT-CT is of special value. Notably we found differences when this general data to different foot regions was itemized. The rate of changed treatment decision was higher in Lisfranc and Chopart joints compared to ankle and subtalar joints. In matter of symptomatic improvement there are influencing factors to consider. The rate could be influenced through a different rate of conservative treatment. Additionally a lack of symptomatic improvement could occur due to an insufficiently performed operation or treatment. Turning toward contingency data a sensitivity and negative predictive value of 1.0 for groups 3 and 4 is remarkable. The reason was that no patient was operated when SPECT-CT was negative. Furthermore the low specificity of group 4 is notable due to a high rate of conservative treatment when SPECT-CT was positive. Accordingly the specificity of group 3 is higher because SPECT-CT was mostly negative in patients with conservative treatment. The values of group 2 are almost identical to the published data of Singh et al. [6]. The sensitivity and negative predictive value of group 1 are the lowest values of the four subgroups. Pagenstert et al. could not verify a different interobserver reliability for different foot regions of SPECT-CT. In the same study the interobserver reliability of SPECT-CT for NC and TMT joint was still significantly higher than for CT and bone scan alone [2].

## 5. Conclusions

SPECT-CT has a high impact on diagnosis and treatment decision and thereby is a beneficial tool in diagnosis of foot and ankle surgery. We could demonstrate that the rate of changed treatment decision and symptomatic improvement was slightly higher in Chopart and Lisfranc joints than in ankle and subtalar joints. Nevertheless the differences were not as high as our hypothesis which could be affirmed unrestricted.

## Supplementary Material

The Supplementary table contains basic data including sex, age, assumed diagnosis, SPECT-CT diagnosis and final treatment for each patient.

## Figures and Tables

**Table 1:** Comparison of groups 1–4.

	Ankle joint (*n* = 31)	Subtalar joint (*n* = 23)	Chopart joint (*n* = 16)	Lisfranc joint (*n* = 16)	Total number (*n* = 86)
Changed diagnosis and treatment	64.5% (20/31)	65.2% (15/23)	75% (12/16)	75% (12/16)	68.6% (59/86)
Surgical treatment	74.2% (23/31)	78.3% (18/23)	56.2% (9/16)	68.8% (11/16)	70.9% (61/86)
Symptomatic improvement	90.3% (28/31)	95.7% (22/23)	93.8% (15/16)	93.8% (15/16)	93% (80/86)

**Table 2:** Clinical diagnosis, SPECT-CT-diagnosis, treatment, and assumed reason of persistent pain of patients without symptomatic improvement.

Patient	Clinical diagnosis	SPECT-CT diagnosis	Treatment	Assumed reason for persistent pain
1	Ankle arthritis, osteochondrosis dissecans tali	Osteochondrosis dissecans tali	Ankle arthroscopy	Degenerative changes

2	Ankle arthritis	Ankle arthritis	Ankle prosthesis	Arthrofibrosis

3	Ankle arthritis	Ankle arthritis	Ankle fusion	Pseudarthrosis

4	Plantar fasciitis, DD subtalar arthritis	Normal	Conservative treatment	Insufficient conservative treatment

5	TN-arthritis, DD-coalitio calcaneonaviculare, adipositas	TN-arthritis, CC-arthritis	Weight reduction	Inconsequent weight reduction

6	Subtalar arthritis, TMT-I-III arthritis	TMT-II-III arthritis	TMT-II-III fusion	Pseudarthrosis
