# Supplementary material for: Influence on Therapeutic Decision Making of SPECT-CT for Different Regions of the Foot and Ankle
Source: Biomed Res Int. 2014 May 18;2014:927576. doi: 10.1155/2014/927576 (PMC4052163; doi:10.1155/2014/927576)
Supplement: Supplementary file 1 — The Supplementary table contains basic data including sex, age, assumed diagnosis, SPECT-CT diagnosis and final treatment for each patient. [file 927576.f1.pdf]

	Pathology site	Sex	Age	Assumed diagnosis	SPECT-CT diagnosis	Final treatment
1	**Ankle joint**	m	56	Ankle arthritis	Ankle and subtalar arthritis	Pantalar arthrodesis
2		m	69	Talus necrosis	Ankle and subtalar arthritis	Pantalar arthrodesis
3		m	26	Non union ankle joint	Non union ankle joint	Rearthrodesis
4		m	46	Ankle arthritis	Moderate ankle arthritis	Conservative treatment
5		m	36	Beginning ankle arthritis	Ankle arthritis	Ankle arthroscopy
6		f	42	Ankle arthritis, Osteochondrosis dissecans tali	Osteochondrosis dissecans	Ankle arthroscopy
7		m	53	Ankle arthritis	Ankle arthritis	Ankle arthrodesis
8		f	68	Painful hindfoot	Ankle arthritis	Conservative treatment
9		m	57	Painful Ankle prosthesis	Aseptic loosening	Ankle arthrodesis
10		m	31	Osteochondrosis dissecans tali	No essential pathology	Ankle arthroscopy
11		m	63	Ankle arthritis	Ankle arthritis	Ankle arthrodesis
12		m	81	Painful hindfoot	Ankle arthritis	Ankle prosthesis
13		m	50	Osteochondral lesion	Moderate ankle arthritis	Conservative treatment
14		f	55	Ankle arthritis	Medial ankle arthritis	Supramalleolar osteotomy
15		m	33	Ankle arthritis	Ankle arthritis	Ankle arthroscopy
16		f	30	Ankle arthritis	Moderate ankle arthritis	Ankle arthrodesis
17		f	47	Ankle arthritis	Ankle arthritis	Ankle prosthesis
18		m	40	Ankle arthritis	Ankle arthritis	Ankle arthrodesis
19		m	40	Ankle arthritis	Ankle arthritis	Ankle arthrodesis
20		f	83	Ankle and subtalar arthritis	Ankle arthritis	Conservative treatment
21		m	44	Painful hindfoot	Ankle arthritis	Conservative treatment
22		m	57	Ankle and subtalar arthritis	Ankle arthritis	Ankle arthrodesis
23		f	65	Painful hindfoot	Ankle arthritis	Ankle arthrodesis
24		f	63	Painful hindfoot	Ankle arthritis	Ankle arthrodesis
25		f	51	Ankle arthritis	Ankle arthritis	Ankle arthrodesis
26		m	33	Osteochondral lesion	Osteochondral lesion	Conservative treatment
27		f	42	Medial ankle arthritis	Osteochondral lesion	Supramalleolar osteotomy
28		m	40	Ankle arthritis	Osteochondral lesion	Ankle arthroscopy
29		m	74	Painful hindfoot	No essential pathology	Conservative treatment

30		f	49	Ankle arthritis	Ankle arthritis	Ankle arthrodesis
31		f	65	Osteochondrosis dissecans tali	No essential pathology	Conservative treatment
32	**Subtalar joint**	f	79	Non union subtalar joint	No essential pathology	Conservative treatment
33		f	34	Plantar fasciitis, DD Subtalar arthritis	No essential pathology	Conservative treatment
34		f	52	Pes varus	Moderate subtalar arthritis	Calcaneal osteotomy
35		m	59	Subtalar Arthritis	Subtalar arthritis	Subtalar arthrodesis
36		f	24	Pes planovalgus	Subtalar arthritis	Double arthrodesis
37		f	41	Painful hindfoot	Subtalar arthritis	Subtalar arthrodesis
38		f	81	Pes planovalgus	Subtalar and TN arthritis	Triple arthrodesis
39		m	55	Ankle arthritis	Subtalar arthritis	Subtalar arthrodesis
40		f	42	Coalitio talocalcaneare	Coalitio talocalcaneare	Resection Coalitio
41		f	53	Subtalar Arthritis	Subtalar arthritis	Subtalar arthrodesis
42		m	43	Pes planovalgus	Coalitio talocalcaneare	Subtalar arthrodesis
43		f	57	Subtalar Arthritis	Subtalar arthritis	Subtalar arthrodesis
44		m	25	Non union subtalar joint	No essential pathology	Conservative treatment
45		f	80	Subtalar Arthritis	Subtalar arthritis	Conservative treatment
46		m	42	Ankle arthritis	Subtalar arthritis	Subtalar arthrodesis
47		m	58	Pes planovalgus	Subtalar and CC arthritis	Triple arthrodesis
48		m	47	Ankle and subtalar arthritis	Subtalar arthritis	Subtalar arthrodesis
49		f	73	Pes planovalgus	Subtalar and TN arthritis	Triple arthrodesis
50		f	77	Painful hindfoot	Moderate subtalar arthritis	Conservative treatment
51		m	46	Subtalar Arthritis	Subtalar arthritis	Subtalar arthrodesis
52		m	45	Subtalar Arthritis after ankle arthrodesis	Subtalar arthritis	Pantalar arthrodesis
53		f	66	Non union subtalar joint	Implant associate reaction	Implant removal
54		f	62	Painful hindfoot	No essential pathology	Subtalar arthrodesis
55	**Chopart joint**	f	16	TN-arthritis, DD Coalitio calcaneonaviculare, Adipositas	TN-arthritis, CC- arthritis	Conservative treatment, weight reduction
56		f	51	Ankle instability	Cyst os naviculare	TN arthrodesis
57		f	42	CC arthritis	No essential pathology	Conservative treatment
58		f	83	Chopart arthritis	Moderate CC arthritis	Conservative treatment
59		f	27	Non union TN joint	No essential pathology	Conservative treatment
60		f	58	TN arthritis after subtalar arthrodesis	TN arthritis	TN arthrodesis

61		f	60	Painful hindfoot	TN arthritis	TN arthrodesis
62		m	36	TN arthritis	TN arthritis	TN arthrodesis
63		f	47	Pes cavovarus	TN arthritis	TN arthrodesis
64		f	56	Pes planovalgus	TN and CC arthritis	TN and NC arthrodesis
65		f	63	Painful hindfoot	No essential pathology	Conservative treatment
66		f	66	Painful hindfoot	Cyst Os naviculare	TN arthrodesis
67		f	68	TN arthritis	No essential pathology	Conservative treatment
68		m	54	Necrosis Os naviculare	Stress reaction Os naviculare	Conservative treatment
69		m	62	Midfoot arthritis	TN arthritis	TN and NC arthrodesis
70		f	64	Ankle arthritis	TN arthritis	TN arthrodesis
71	**Lisfranc joint**	m	53	Non union TMT I-III joint	Non union TMT I-III joint	Rearthrodesis
72		m	46	TMT arthritis	No essential pathology	Conservative treatment
73		f	71	Pes planus	TMT I arthritis	Conservative treatment
74		f	71	Midfoot arthritis	TMT II+III arthritis	TMT I-III arthrodesis
75		f	63	Subtalar arthritis, TMT-I-III arthritis	TMT-II-III-arthritis	TMT-II-III-fusion
76		f	68	Midfoot sprain	TMT I-III arthritis	TMT I-III arthrodesis
77		f	82	Lisfranc arthritis	TN and TMT arhtritis	TMT I-III arthrodesis
78		m	35	Painful midfoot	TN and TMT arhtritis	Conservative treatment
79		f	58	Midfoot deformety	TMT II arthritis	TMT II+III arthrodesis
80		f	77	Painful midfoot	TN and TMT arhtritis	TMT I-III arthrodesis
81		f	56	Non union metatarsal II	TMT II arthritis	TMT II arthrodesis
82		f	52	Non union TMT I-III joint	Implant associate reaction	Implant removal
83		m	68	TMT arthritis	TMT II+III arthritis	TMT II+III arthrodesis
84		f	52	Painful midfoot	TMT I arthritis	Conservative treatment
85		f	55	TMT arthritis	TMT II+III arthritis	Conservative treatment
86		f	63	Painful midfoot	TMT II+III arthritis	TMT I-III arthrodesis

Supplementary table **Basic data**

The clinical diagnosis, diagnosis of SPECT-CT and final treatment are demonstrated
